# Does migratory distance affect fuelling in a medium-distance passerine migrant?: results from direct and step-wise simulated magnetic displacements

**DOI:** 10.1242/bio.014779

**Published:** 2016-02-16

**Authors:** Mihaela Ilieva, Giuseppe Bianco, Susanne Åkesson

**Affiliations:** 1 Centre for Animal Movement Research, Department of Biology, Lund University, Ecology Building, Lund SE-223 62, Sweden; 2 Institute of Biodiversity and Ecosystem Research, Bulgarian Academy of Sciences, 2 Gagarin str., Sofia 1113, Bulgaria

**Keywords:** Bird migration, Diurnal migrant, Dunnock, Food intake, Geomagnetic field, *Prunella modularis*

## Abstract

In birds, fat accumulation before and during migration has been shown to be endogenously controlled and tuned by, among other factors, the Earth's magnetic field. However, our knowledge about the influence of the geomagnetic field on the fuelling in migrating birds is still limited to just a few nocturnally migrating passerine species. In order to study if variations of the magnetic field can also influence the fuelling of both day- and night-migrating passerines, we caught first-year dunnocks (*Prunella modularis*) and subjected them to three magnetic field conditions simulated by a system of magnetic coils: (1) local geomagnetic field of southern Sweden, (2) magnetic field corresponding to the centre of the expected wintering area, and (3) magnetic field met at the northern limit of the species' breeding distribution. We did not find a difference in mass increase between the birds kept in a local magnetic field and a field resembling their wintering area, irrespectively of the mode of magnetic displacement, i.e. direct or step-wise. However, the dunnocks magnetically displaced north showed a lower rate of fuelling in comparison to the control group, probably due to elevated activity. Compared with previous studies, our results suggest that the fuelling response to magnetic displacements during the migration period is specific to the eco-physiological situation. Future studies need to address if there is an effect of magnetic field manipulation on the level of migratory activity in dunnocks and how widespread the influence of local geomagnetic field parameters is on fuelling decisions in different bird species, which have different migratory strategies, distances and migration history.

## Abstract (precis)

**Summary:** Fuelling rate in migrating dunnocks, a predominantly diurnal migratory songbird is influenced by a manipulated magnetic field when the birds are magnetically displaced north, but not south, in autumn.

## INTRODUCTION

Bird migration is a highly complex phenomenon involving phenotypic adaptations of morphology, behaviour and physiology, allowing individual birds to move over large distances, sometimes thousands of kilometres, in response to seasonal changes in the environment (Åkesson and Hedenström, 2007). Many of the processes connected with bird migration have been shown to be endogenously controlled, such as fat accumulation, migration distance, timing and direction of migration (Berthold, 1996; Liedvogel et al., 2011). However, the genetic program encoding phenotypic responses needs to be in constant interaction with the environment in order to fulfil its role. Various external cues fine-tune the ecophysiological responses and migratory behaviour en route, and thereby support the individual bird to accomplish the migration journey successfully. Such cues might for example be the photoperiod, information from the geomagnetic field, stellar cues, temperature and interactions with conspecifics (Gwinner, 1996; Chernetsov et al., 2004; Hansson and Åkesson, 2014).

A common feature of the endogenous program of migrating birds is the accumulation of fuel reserves, mostly in the form of unsaturated fatty acids (Walker, 1964). The fat reserves allow birds to fly long distances, but excessive fat loads can also have disadvantages, such as increased flight costs (Alerstam and Lindström, 1990; Hedenström and Alerstam, 1997), and reduced escape performance (e.g. Hedenström, 1992; Kullberg et al., 1996). In some instances, such as crossing large ecological barriers, carrying large amounts of fuel is necessary, because of limited possibilities to refuel, while it is more favourable to migrate with smaller fuel stores when refuelling opportunities are present during the migration route (Kaiser, 1992; Berthold, 1993; Ilieva and Zehtindjiev, 2005). Smaller fuel stores may further facilitate efficient climbing flight (Hedenström and Alerstam, 1992).

The geomagnetic field is an important orientation cue for migratory birds. The use of magnetic information in avian migratory orientation was proven beyond doubt in the 1960s and has been studied extensively in this respect since then (Merkel and Wiltschko, 1965; Wiltschko and Wiltschko, 1972, 1995; Lohmann and Johnsen, 2000; Mouritsen and Ritz, 2005). Geomagnetic information has further been shown to be used in defining latitude in migrating birds (Fischer et al., 2003). However, until very recently the use of a magnetic map in birds has not been clearly supported by experimental data as it was for hatchling sea turtles and spiny lobsters (Lohmann et al., 2001; Boles and Lohmann, 2003). The first evidence of the use of a magnetic map in birds was reported by Kishkinev et al. (2015), showing that Eurasian reed warblers (*Acrocephalus scirpaceus*) change course and orient towards their breeding grounds in spring after virtual magnetic displacement east of their site of capture. The results suggest the use of magnetic cues for positioning at least in some songbirds, but it should be generalized with caution including other bird taxa as for example homing pigeons, tubenoses and gulls where the olfactory map sense hypothesis so far has received more experimental support (Gagliardo et al., 2009, 2013; Wikelski et al., 2015).

Comparatively less attention has been given to the influence of magnetic information on fuelling decisions of migratory birds (Fransson et al., 2001; Kullberg et al., 2003, 2007; Henshaw et al., 2008; Boström et al., 2010, 2012) compared to compass orientation and navigation. Fransson et al. (2001) showed that young migratory thrush nightingales (*Luscinia luscinia*) in Sweden exposed to a simulated magnetic field of northern Egypt in autumn, corresponding to a location before a large ecological barrier, the Sahara desert, increased weight significantly more than the control group kept in the local magnetic field in Sweden. In a later experiment the same research group showed that the magnetic field could also influence fuelling in a short to medium distance migrant, the European robin (*Erithacus rubecula*) (Kullberg et al., 2007). In this study the European robins, which were gradually displaced step-wise to a local geomagnetic field corresponding to that of their wintering area in Spain, kept a constant weight during the experimental period, while individuals kept in the local magnetic field in Sweden increased their fuelling rate. Repeating the same treatments, however, with both species early and late in the season yielded different outcomes (Kullberg et al., 2003, 2007; Henshaw et al., 2008). In thrush nightingales, a difference between the control and experimental group was observed only in birds tested early in the migration season, while in European robins the effect was observed only when birds were tested later in the season. These results show a strong seasonal effect on the physiological process of fuelling, depending on the migration distance and strategy of the species studied, suggesting specificity in the evolution of the migration fuelling phenotypes. The outcomes of the comparative experiments with thrush nightingales with simulated multiple or single-step magnetic displacements to Egypt were very similar (Henshaw et al., 2008), suggesting a hard-wired response to intensity and inclination of the external magnetic field. The majority of experiments studying fuelling in songbirds as an effect of magnetic field manipulations have so far been studied in a single laboratory. In this study, performed in southern Sweden, we have focused our work on a short or medium distance avian migrant, the dunnock, *Prunella modularis* (Linnaeus 1758), studying how this species respond to a single-step magnetic field displacement to their wintering grounds in southwestern Europe. In particular, we investigated if the level of fuel reserves was affected by changes of the geomagnetic field parameters field intensity and inclination angle, expected to be met at the winter destination in southwestern Europe.

Dunnocks are small passerine birds breeding throughout Europe and in parts of western Asia (Cramp, 1988). Dunnocks are medium and short-distance migrants in the northern and central parts of their European breeding range. Based on ringing recoveries, Fransson and Hall-Karlsson (2008) showed that dunnocks ringed in Sweden migrate towards the southwest in autumn, passing Germany and France in October and November, respectively and spending the winter mainly in southwestern France. The numbers of dunnocks caught at a wintering site in northwestern Spain, close to the border to France, show a clear increase in numbers during October and November, reaching maximum numbers in the end of November (Pons, 2001).

Overall, the dunnock, as a short- to medium-distance migrant, does not show a large increase in body mass during migration as trans-Saharan passerine migrants such as the thrush nightingale do (Fransson et al., 2001). The mean weight of immature dunnocks caught during autumn migration increase slightly the further south they were caught – from 18.45 g [corresponding to mean fat score 2.7 (range 0-4)] in southwest Sweden (our personal observations), 19 g in southwest Germany (Kaiser, 1992) to 19.1 g at the island of Capri in Italy (Hjort et al., 2006). The mean weight of the Scandinavian dunnocks wintering in France most probably is similar to the mean mass of 17.4 g recorded during February and March in Italy (Hjort et al., 2006).

Several studies suggest that dunnocks are able to detect and use the earth's magnetic field as an orientation cue during migration. In orientation experiments during spring migration, Bingman and Wiltschko (1988) showed that when magnetic north was artificially shifted 120° to ESE, the birds showed a corresponding shift in their orientation. Since the experiments were done with accessibility to celestial cues under a clear sky, the authors concluded that the earth's magnetic field served as a primary directional reference for the migratory dunnocks at the time of sunset. Support for the use of a magnetic compass in dunnocks has also been provided by experiments by Åkesson et al. (2015). In these experiments dunnocks tested in autumn under simulated overcast conditions without access to celestial cues, oriented south, close to the expected migratory direction both in the control condition as well as after cue-conflict exposure. In the same study, individuals tested under a clear sky in a vertical magnetic field showed an axial response in agreement to what was observed in the study by Bingman and Wiltschko (1988).

To study if variations of the magnetic field can also influence the fuelling of this species, we caught first-year migratory dunnocks in southern Sweden and kept them in three magnetic field conditions: (1) the local geomagnetic field, (2) a simulated magnetic field corresponding to the centre of the expected wintering area, and (3) the magnetic field met at the northern limit of the species' breeding distribution. The latter condition was used since, a northern displacement, extending the apparent migration route in autumn to the double, has previously been shown to trigger increased fuelling in Northern wheatears *Oenanthe oenanthe* (Boström et al., 2012). Hence, in the two latter magnetic conditions, we simulated different distances to the wintering area. We expected that the further north the birds were from their wintering area the higher fuelling rates they would exhibit. We further hypothesized that birds exposed to a magnetic field resembling the wintering area would show reduced, if any, mass increase because the magnetic field would signal arrival at their destination. However, we expected to observe only small variations, since dunnocks migrating from southwestern Sweden in autumn do not need to pass large ecological barriers en route, and therefore carry limited fuel reserves (Pons, 2001). In addition to the magnetic field conditions outlined above, we compared fuelling responses for individual birds to a direct and gradual step-wise magnetic displacement to the expected wintering destination.

## RESULTS

There was no difference in temperature experienced by the birds between treatments during the 12 days of experiments (Table 1). The mean temperature in the sheds was slightly higher during the first compared to the second experimental period (mean temperature±s.d. at 9:00 am were 12.8±2.4°C and 11.5±3.3°C for experiment 1 and experiment 2, respectively).

**Table 1.**
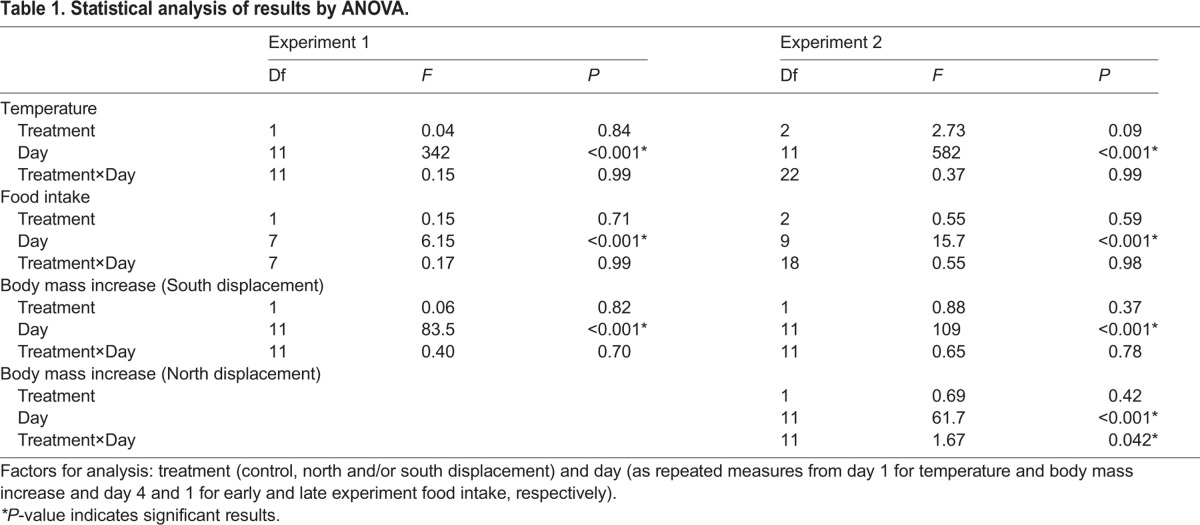
**Statistical analysis of results by ANOVA.**

**Fig. 1.**
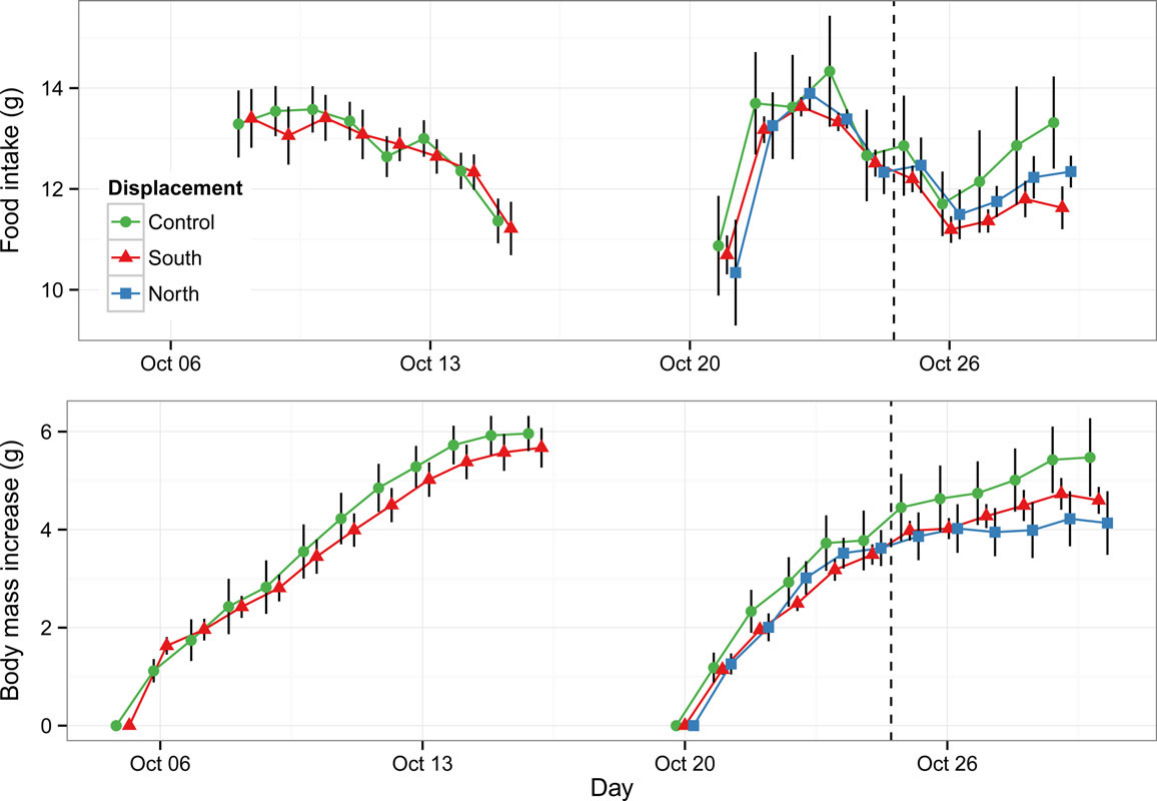
**Food intake and body mass increase from day 1 in migratory dunnocks in two 12-day magnetic displacement experiments during autumn 2014.** In the first experiment birds were displaced to the southern destination area (Control *n*=11; South *n*=12) from day 1 (Oct 5). In the second experiment birds were displaced both to the southern destination area and the northern limit of species distribution range (Control *n*=6; South *n*=8; North *n*=7). Moreover, the magnetic field was changed every day during 6-day period (until the dashed line) and then kept to a constant value. The geographical positions corresponding to the simulated magnetic displacements were Sweden (Control), southern France (South) and northwestern Russia (North) as reported in Fig. 2 and Table 2. Data represented as mean±s.e.m.

There was no difference in food intake between control and treated birds in either experiment (Table 1). In experiment 1, with an increase of the birds' body mass, the food intake dropped from 13.3±2.1 g to 11.3±1.6 g, while in experiment 2 the mean food intake was lowest in the beginning and highly variable between individuals toward the end of the experimental period (Fig. 1). It should, however, be kept in mind that in experiment 1 the recording of food intake started a few days later than in experiment 2, and thus the food intake in the first few days therefore cannot be compared between both experiments.

In experiment 1, there was no difference in initial body mass between the birds in the control and the south displacement group (one-way ANOVA, *F*_1,21_=0.61, *P*=0.44) with starting body mass of 19.8±1.0 g and 19.4±1.5 g for the control and south displacement group, respectively. Neither was there any difference in initial body mass in experiment 2 between the birds in the two treatments (North: *F*_1,11_=1.98, *P*=0.19; South: *F*_1,12_=2.6, *P*=0.13) with control birds being 22.7±0.8 g, south 23.6±1.0 g and north 21.9±1.3 g. All included birds increased in body mass during both experiments, but while there was no difference between groups over time in experiment 1, there was a significant difference between the control and the north displacement group in experiment 2, but not for the south displaced group (Table 1).

## DISCUSSION

When subjected to magnetic displacements to their wintering areas in southwestern France (Fransson and Hall-Karlsson, 2008), the experimental first-year dunnocks did not show a difference in fuelling compared to the control birds kept in the local geomagnetic field in southern Sweden, irrespectively of the type of magnetic displacement, i.e. direct or step-wise. These results are in contrast to the outcome of previous experiments with thrush nightingales, where a single-step magnetic displacement to Egypt lead to a significant increase in body mass similar to what was observed when a multi-step magnetic displacement was applied (Fransson et al., 2001; Henshaw et al., 2008). However, our study has some important differences compared to the study of Henshaw et al. (2008), which hamper their direct comparison. The applied magnetic fields in the above-mentioned studies and our study corresponded to different migration stages in the two species, a staging area prior to a Sahara crossing for the thrush nightingales and arrival at the wintering destination for the dunnocks in our study. According to optimal bird migration theory, birds are expected to accumulate fat in a strategic way depending on the possibilities to refuel en route (Alerstam and Lindström, 1990; Hedenström and Alerstam, 1997). In the case of the thrush nightingale, which is a long-distance tropical migrant, the increase of fuel loads before the crossing of major ecological barriers such as the Mediterranean Sea and the Sahara desert is of crucial importance and likely under strong selection pressure. The dunnock on the other hand, is a short to medium-distance migrant, flying mostly over land during the daytime where suitable stopover sites presumably are widespread and may be easily detected. Hence, it is probably less important for dunnocks to accumulate fuel stores at a particular part of their migratory journey and this might explain the weaker connection between the magnetic field and fuelling in dunnocks in contrast to the long-distance migrants studied so far (Fransson et al., 2001; Boström et al., 2010). When compared with another short to medium-distance migrant, the European robin (Kullberg et al., 2007), which has similar habitat preferences as the dunnock, the later species reacts to ‘displacements’ not via change in its food intake but with other mechanisms, most probably changes in the activity level. This suggests that there can be a variation in responses to magnetic field information even in species with very similar habitat choice and migration timing and distance.

The mean body mass reported for Fenno-Scandinavian dunnocks caught along their migratory route in autumn is around 19 g, but birds with body weight as high as 23-25 g have been recorded in Finland and on the Helgoland island in Germany (Cramp, 1988 and references therein). This suggests limited fat levels overall in migrating dunnocks during autumn migration. The mean maximum weight of our experimental birds reached the considerably higher value of 26.7 g (Table S1; corresponding to fat score 5), probably showing the maximum fattening capacity for dunnocks. This observation is further supported by the fact that despite the unlimited access to food, at the end of both experiments, our birds reached a plateau and stopped increasing in weight.

The lack of response to both types of magnetic displacement, i.e. gradual or direct, to the wintering areas also distinguish dunnocks from the only other short-distance migrant studied in this respect so far, the European robin (Kullberg et al., 2007). While the European robins subjected to a step-wise magnetic displacement to their wintering areas later in the migration period kept a constant weight throughout the experiment, the dunnocks from the control and the experimental group in our study both increased their body mass. Moreover, in our experiments we tried for the first time to subject a medium- to short-distance migrant to a direct magnetic displacement to the wintering grounds. However, irrespective of mode of displacement we could not observe any differences in the fuelling between the control and experimental birds magnetically displaced south, suggesting no clear effect of type of magnetic displacement.

A significantly lower rate of fuelling was observed in the group which was magnetically displaced north in comparison with the control birds. Since there was no statistically significant difference in the food intake between the three groups (south, north and control) in the second experiment, we might speculate that birds subjected to the northern magnetic field most likely were more active than the control birds. Unfortunately, we did not have the possibility to record the birds' behaviour in the cages during the experiments and could not quantify their activity. The reduced increase in body mass of the northern group shows that dunnocks can react to for them relevant values of the magnetic field, in this case possibly by increasing their activity when noticing that they are too far north relative to the destination area. Since this experiment was performed late in the migration season, in the end of October, the dunnocks were probably additionally pressed by the magnetic values corresponding to much more northern position than normal for this time of season. Increased mass, as a response to simulation of more ‘northern’ magnetic values, was observed in the experiments by Boström et al. (2010) studying first-year northern wheathers (*Oenanthe oenanthe*). In this case a higher increase in body mass was observed in the experimental group, but again without any noticed difference in food intake between the two groups. However, in the experiments by Boström et al. (2010) the birds were displaced outside the migration route of the studied population, which might be one reason for the difference in results. When delayed on migration, long-distance migrating warblers caught in Sweden are able to speed up their migration, as the amount of subcutaneous fat is positively related to the speed of migration (Fransson, 1995, Bensch and Nielsen, 1999). However, in dunnocks and also in northern wheathers from the above-mentioned study (Boström et al., 2010), the difference in body mass between the control and experimental birds seems to come not from higher food intake of one of the groups, but rather from different activity levels.

**Fig. 2.**
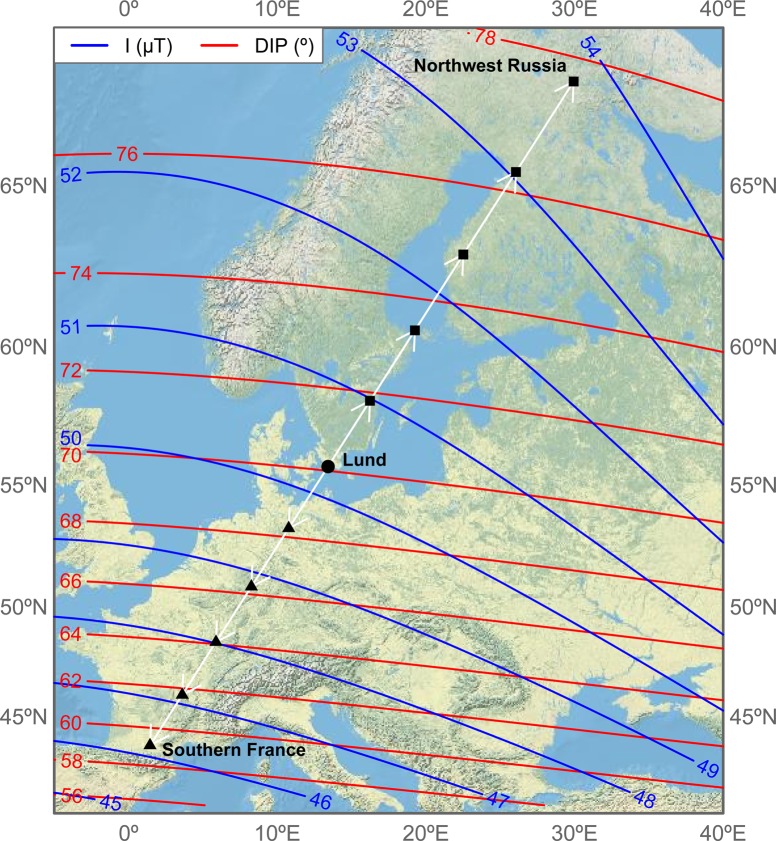
**Map showing the experimental location and step-wise magnetic displacements.** The experiments were performed in Lund (Sweden) and the birds exposed to 5-day simulated stepwise displacements (white arrows) toward the final destinations in southern France (population-specific wintering grounds) and the northwestern Russia (at the northern limit of European species distribution). All displacements are 319 km long and lie on the rhumb line passing through the experimental site. Map is in Mercator projection and also reports magnetic intensity (I) and inclination (DIP) isolines as obtained by the eleventh International Geomagnetic Reference Field model (IGRF-11) for the 20th of October 2014. Locations' coordinates and magnetic values are given in Table 2.

According to the Swedish Ringing Atlas (Fransson and Hall-Karlsson, 2008) dunnocks ringed in Sweden have a mean maximum autumn migration speed of 69 km/day. Thus, they will cover the distance of our magnetic displacement of nearly 1600 km in 23 days. In our experiments, as well as in most of the other similar experiments, the change in the magnetic field was direct (experiment 1) or much faster (the final destination reached in five days in the step-wise displacement; experiment 2) in comparison with the natural situation experienced by the birds. Using the IGRF-11 model for the 20th of October 2014 we have calculated that the maximum daily changes in magnetic field (mean±s.d.) for birds migrating along the rhumb line, defined as in Fig. 2, will be much smaller (South: Intensity=176±18 nT/day; Inclination=0.47±0.06°/day; North: Intensity=150±4 nT/day; Inclination=0.33±0.03°/day) than in the five days magnetic displacement in our step-wise experiment (South: Intensity=822±83 nT/day; Inclination=2.20±0.32°/day; North: Intensity=700±71 nT/day; Inclination=1.52±0.13°/day). However, the total change in magnetic intensity and especially the inclination is larger for the birds displaced south in comparison with the ones displaced north (South: Intensity=4100 nT; Inclination=11°; North: Intensity=3500 nT; Inclination=7.6°). Therefore, we do not believe that the shorter time of the experiments and consequently the faster change of magnetic field values is the reason for the similar fuelling rate between the control and the south ‘displaced’ experimental birds, but not for the north ‘displaced’ birds.

The results of our study with dunnocks compared to the studies with several other species of migrants (Fransson et al., 2001; Kullberg et al., 2007; Henshaw et al., 2008; Boström et al., 2010), suggest that the fuelling response to magnetic displacements during migration periods might be species-specific and dependent on the eco-physiological situation the birds meet on migration. In our study we did not find an effect of magnetic displacements on the food intake. We further found no difference in fuelling between the dunnocks magnetically displaced to their wintering grounds as compared to the controls. The lower rate of fuelling in dunnocks displaced magnetically north, compared to the controls, might have been triggered by a change in locomotor activity but it needs to be experimentally tested in the future. Our results stress the need to perform more experiments to study in what ways and how widespread the influence of the magnetic field are on fuelling decisions in different bird species, focusing on species with different migratory strategies, distances and migration history.

## MATERIALS AND METHODS

### Experimental birds and keeping conditions

We captured first-year dunnocks using mist-nets near Stensoffa Ecological Field Station (55°41′N 13°26′E) in southwestern Sweden in October 2014. At capture all birds included in this study (2×24) had finished their post-juvenile moult and had fat scores between 1 and 3 (fat score 4 for one bird), according to the visual scale for fat classification of Pettersson and Hasselquist (1985) and extended by tree scales to 0-9 at Falsterbo Bird Observatory (Sjöberg et al., 2015). Hence, we selected experimental birds that were in migratory condition. Birds were kept indoors in individual cages (80×50×71 cm) for several days before the start of the experiments, and were provided with food and water *ad libitum*. Permissions for this work were given by the Malmö/Lund Ethical Committee for Scientific work on animals (no. M33-13), the Swedish Board of Agriculture for housing facilities (Dnr. 31-2295/09), the Swedish Nature Protection Agency and the Swedish Ringing Centre (No. 440) to S.Å.

During the experiments, birds were placed in individual nonmagnetic cylindrical cages (550 mm diameter, 700 mm height) in six wooden sheds (15 m^2^ internal surface and 3.9 m maximum high) with semi-transparent plastic roofs (Fig. S1). In each shed four cages were placed in the middle of an electromagnetic coil allowing manipulation of the magnetic field inside the coil (Fig. S1). The cages were made from non-transparent grey (PVC) plastic and had a PVC perch in the middle and PVC net on the top of the same colour. The inner walls of the cages were covered with polyester foam to provide acoustic isolation without polarising the reflected light. Four trays for placing feeders (or water dispensers) were accessible from underneath each cage. During the experiments the birds were provided daily, at 9 AM local summer time (+2:00 UTC), with fresh water and mealworms (*Tenebrio molitor*) and the amount of food eaten was estimated by weighing the remaining food. In the first experiments the food was weighted from day 4, while in the second it was done from the beginning of the experiment. The weight of individual birds was recorded each day at 5 PM local summer time (+2:00 UTC) by using electronic scales (Precisa BJ410C; Precisa Gravimetrics AG, Dietikon, Switzerland) situated under each cage and connected with the perch (Fig. S1). Birds were carefully checked if eating in the experimental cage in the mornings and afternoons until all were recorded to be eating. Most of the birds started eating during the first day of the experiment, but a few started somewhat later (Table S1). All birds started to eat in the experimental cages and were therefore included in the study. In each shed the temperature was measured and registered continuously throughout both experiments using a Comet T3610 remote sensors device (0.1°C resolution) (COMET SYSTEM, s.r.o., Roznov pod Radhostem, Czech Republic). Temperature values were automatically acquired every minute trough a local Ethernet network and hourly averaged before analysis.

### Magnetic coil system

The magnetic field in each shed was modified using a Merritt three square-coil system (Merritt et al., 1983). The nine square coils were made of three different dimensions. The largest coils set (side length: 1620 mm) were aligned to control the vertical direction of the magnetic field; the two smaller sets were sized to fit inside the larger coils and aligned to control the north-south and east-west directions, respectively (Fig. S1). The coils were realised using U-shaped aluminium ranks wrapped with insulated copper wire and each triplet of coils aligned in the same directions were feed in series with one of the channels of an Hameg HMP4040 DC power supply (current accuracy ±1 mA; HAMEG Instruments GmbH, Mainhausen, Germany). The power supplies were located in a shielded cabinet several meters away from the closest shed. Before the experiments were initiated, the magnetic field values were set using a three-axis digital magnetometer (Honeywell HMR2300; Honeywell International Inc., Plymouth, UK). To be sure that all the birds were exposed to similar magnetic field conditions, particular attention was made in the cage arrangements. To assure near to homogenous magnetic conditions between cages in the set-ups, the cages were positioned on a 1200×1200 mm plywood non-magnetic table with height-adjustable PVC feet. The heights of the feet were then adjusted to have the top of the perch (i.e. where birds are spending most of the time) aligned with the middle of the three vertical coils. Furthermore, multiple measurements where performed at the top of each perch to ensure that the magnetic field was within the 1% tolerance level between the cages of the same shed.

### Experimental treatments

We performed two consecutive experiments with 24 dunnocks in each group, starting on 5 and 20 October, respectively and lasting 12 days each. Birds in both experiments were randomly assigned for different types of treatments. In the first experiment 12 dunnocks were exposed to the magnetic field corresponding to their expected wintering grounds in southern France (Fransson and Hall-Karlsson, 2008), and 12 birds were used as controls and kept in the local magnetic field (Fig. 2, Table 2). In the second experiment we divided the birds to three groups, eight individuals each. One group was kept in control conditions in the local magnetic field while the other two groups were gradually ‘displaced’ to the south and north, respectively (Fig. 2, Table 2). We used different modes of magnetic displacements in the two experiments. In the first experiment, the birds from the experimental group were directly subjected to the magnetic field of their wintering area at the start of experiment. In the second experiment, the magnetic field was changed gradually. In the first day all birds were exposed to the local magnetic field, and then each day at noon the magnetic fields of the experimental groups was changed in successive steps for five days until day 6 was reached and the values of the wintering grounds in France, or the corresponding shift to the northern field in Lapland were reached (south and north group, respectively; Table 2). After the final values were reached, the birds were kept in these magnetic fields for six more days. All used values of the magnetic field were calculated using the Fortran code of the eleventh International Geomagnetic Reference Field model (IGRF-11, Finlay et al., 2010). After the end of each of the experiments the dunnocks were released into the wild.

**Table 2.**
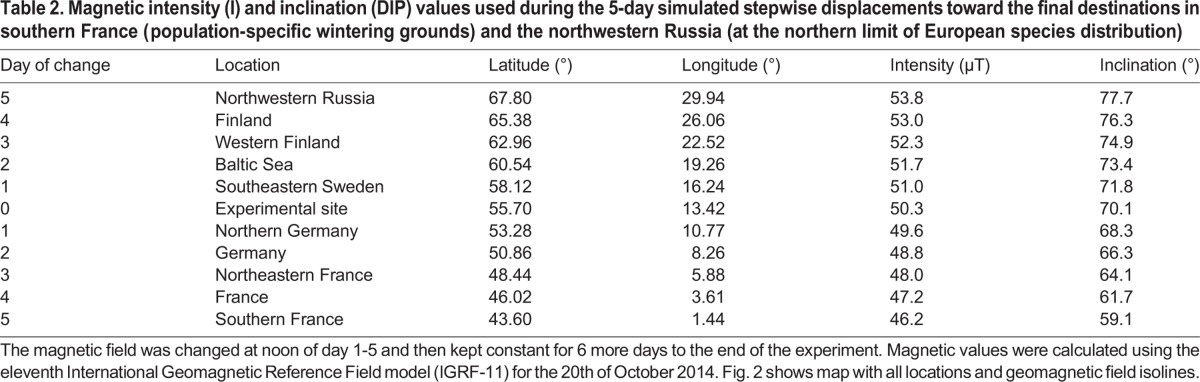
**Magnetic intensity (I) and inclination (DIP) values used during the 5-day simulated stepwise displacements toward the final destinations in southern France (population-specific wintering grounds) and the northwestern Russia (at the northern limit of European species distribution)**

### Data analysis

One bird in experiment 1 and three birds in experiment 2 did not gain but lose body mass during the first 1-4 days. In experiment 1, the bird was from the control group, while in experiment 2 they were both from the control group (two) and the North displacement treatment (one) (Table S1). These birds started eating in the cage much later than the majority of the experimental birds. During the food replacement/weighting we noticed that in at least two of these cases the birds were substantially more active the first 1-2 days compared to the other birds, and this probably affected their fuelling performance and mass gain. For this reason those four birds were removed from the analysis. All the plots and statistical tests were done in R version 3.1.1 (R Core Team, 2014).

## Supplementary information

Supplementary information available online at http://bio.biologists.org/lookup/suppl/doi:10.1242/bio.014779/-/DC1
